# Embedding patient and public involvement in dementia research: Reflections from experiences during the ‘Journeying through Dementia’ randomised controlled trial

**DOI:** 10.1177/14713012221106816

**Published:** 2022-06-07

**Authors:** Jules Beresford-Dent, Kirsty Sprange, Gail Mountain, Clare Mason, Jessica Wright, Claire Craig, Linda Birt

**Affiliations:** Centre for Applied Dementia Research, 1905 University of Bradford, Bradford, UK; Nottingham Clinical Trials Unit, 6123 University of Nottingham, Nottingham, UK; Centre for Applied Dementia Research, 1905 University of Bradford, Bradford, UK; Clinical Trials Research Unit, The University of Sheffield, Sheffield, UK; Lab4Living, 7314 Sheffield Hallam University, Sheffield, UK; School of Health Sciences, 6106 The University of East Anglia, Norwich, UK

**Keywords:** patient and public involvement and engagement, dementia, research

## Abstract

**Background:**

The involvement of people with a diagnosis of dementia in patient and public involvement and engagement (PPIE) in research is an emerging field in the delivery of studies. Researchers need to understand and use the learning derived from various projects so that this growing body of knowledge can be applied in future research.

**Objective:**

To embed PPIE throughout a randomised controlled trial of a psychosocial intervention called Journeying through Dementia. We identify and discuss the approaches to involvement that worked well and those where improvements were indicated.

**Design:**

The Guidance for Reporting Involvement of Patients and the Public Short Form (GRIPP2-SF) is used to describe and critically appraise the approaches taken and the impact of PPIE involvement upon study processes, the study team and those people with dementia and their supporters who acted as advisors.

**Findings:**

The involvement of people with a diagnosis of dementia and supporters as study advisors improved the accessibility and relevance of the research for people living with dementia. It also highlighted issues that researchers may have otherwise overlooked. Successful engagement of people with dementia and their supporters in the study was associated with staff skills and particularly use of techniques to scaffold meaningful involvement, as well as participants’ memory and cognitive capacity. However, embedding robust and meaningful involvement processes required significant time and resources.

**Discussion:**

We propose that certain research processes need to be adapted to be accessible and appropriate for people living with dementia. Recruitment of PPIE advisors needs to reflect population diversity. There also needs to be greater parity of voice between people with lived experience of dementia and researchers. These steps will increase the impact of PPIE in research and improve the experience for those who volunteer to be PPIE advisors.

## Background

Funders, such as the UK National Institute for Health Research (NIHR) emphasise the value of patient and public involvement and engagement (PPIE) in the development, delivery and dissemination of research and demand that all studies evidence this (National Institute for Health and Care Excellence, 2021). Moreover, it is increasingly necessary to fully describe PPIE in published research outputs using reporting tools such as the GRIPP2 (Staniszewska et al., 2017). Integrity, quality, impact and relevance are just some of the benefits identified through involving those with lived experience (Miah et al., 2019; Poland et al., 2019). PPIE is therefore considered integral to good research design (Stevenson & Taylor, 2019). Guidance now exists on best practice to facilitate PPIE in the design and conduct of research including for people living with dementia (DEEP; National Institute for Health and Care Excellence, 2021).

However, it has been noted that undertaking meaningful PPIE can be challenging for both researchers and those who volunteer as PPIE advisors (Jackson et al., 2020), for example, those with lived experience may question researcher preferences or decisions (Rose & Kalathil, 2019). The power imbalance that can exist within healthcare and research contexts can result in service users being rendered unable to influence research design, implementation and outcome (Novek & Wilkinson, 2019). Therefore approaches to public involvement need to be relevant, accessible and support meaningful engagement (Burton et al., 2019). When working with people living with dementia this may involve managing individual expectations of cognitive capacity, including those of the researcher (Waite et al., 2019), whilst also empowering and valuing the voice of lived experience (Birt & Poland, 2021). Alzheimer Europe’s position on PPIE is one of inclusivity, and encouraging engagement such as identifying research priorities, interpretation of research findings and dissemination (Gove et al., 2018). Identifying and applying methods of PPIE that are acceptable and understandable to people living with dementia, both those living with a diagnosis of dementia and their family supporters, is therefore vital to improve the depth, delivery and utility of dementia research (Burton et al., 2019).

Journeying through Dementia is a psychosocial intervention designed for those diagnosed with mild dementia. It aims to equip individuals with the knowledge, skills and understanding to be able to self-manage and maintain independence and meaningful participation, thereby improving mastery, wellbeing and life satisfaction (Wright et al., 2019). PPIE was embedded throughout the entire Journeying through Dementia research programme from inception and design of the intervention (Mountain & Craig, 2012), through to feasibility testing (Sprange K et al., 2015) and the definitive randomised controlled trial involving 480 people with a diagnosis of dementia and 350 supporters (Wright et al., 2019) The terminology of ‘carer’ or ‘supporter’ was debated by the study team including PPI members who concluded that for people living with mild dementia the term ‘carer’ was inappropriate. We therefore refer to supporters throughout the trial to describe informal carers such as partners, relatives or neighbours. This paper describes our experience of PPIE in the Journeying through Dementia randomised controlled trial. Through reporting our experiences, we aim to highlight when and how PPIE strengthened our research, and also the challenges we encountered whilst endeavouring to deliver meaningful approaches towards PPIE.

## Methods

The GRIPP2 short form format for reporting involvement of patients and the public is used to report and critically reflect upon the PPIE processes and outcomes (Staniszewska et al., 2017).

### Establishing and facilitating patient and public involvement and engagement in the trial

Our approach to PPIE, was informed by NIHR INVOLVE guidelines (National Institute for Health and Care Excellence, 2021), the Dementia Engagement and Empowerment (DEEP) project (DEEP), the work of the Scottish Dementia Working Group (Alzheimer Scotland Action on Dementia, 2020), and the experiences of study team members. Our aims were to:

• Create opportunities for meaningful involvement of people living with dementia and family supporters in the design and delivery of the study and in the dissemination of results.
• Increase the relevance and accessibility of the research to people living with dementia and other members of the public.
• Create relevant, accessible and useful outputs from the study for people affected by dementia.

To support the aims of PPIE engagement in the trial the research team agreed and upheld a number of ‘guiding principles’ to inform the planning and execution of involvement activities, see Box 1.

Box 1 Guiding principles for PPIE involvement in the Journeying through Dementia trial

• All PPIE advisors compensated for their time in line with UK NIHR INVOLVE guidance (NIHR INVOLVE).
• A ‘you said, we did’ approach regarding how advice was used and taken forward.
• Use of best practice accessibility guidance (DEEP), for example, avoidance of jargon, acronyms and academic language.
• Meeting venues selected in consultation with PPIE advisors to ensure accessibility, for example, layout, transport, low noise levels. Provision of wayfinding advice to venues, for example, maps and instructions. Taxis provided if required while at the same time being mindful of offering support rather than becoming paternalistic (Roberts et al., 2019).
• Venue preparation to ensure a dementia friendly layout, provide additional signage and ‘meet and greet’ to help direct people.
• Inclusion of regular breaks during meetings as well as time and space to engage and respond to materials or discussion topics.
• Use of aide-memoires in all encounters with PPIE advisors, for example, flipcharts posted up in meeting rooms to remind members of the aims of the study and of the specific meeting, providing verbal or written updates on study progress.
• For all PPIE meetings content limited to one item per meeting, for example, input to documentation, dissemination activity.
• For Trial Steering Committee meetings, papers sent in hard copy well ahead of the meeting with highlighted sections and extensive annotations to explain reports and figures for specific consideration.

For any study it is essential that appropriate funding is allocated to PPIE activity for reimbursement or payment of time as well as for associated costs such as travel or equipment (NIHR INVOLVE). At the outset a budget was identified for PPIE for the Journeying through Dementia trial although this needed to be increased over time by moving finance from other budget lines within the overall grant allocation to meet the costs of additional activities. We provided regular updates to the funders about PPIE activities and their impact.

Applying an appropriate level of expertise towards PPIE engagement is recognised as being essential. A researcher with previous experience of facilitating engagement of people living with dementia in research (the PPI Lead), was responsible for identifying PPIE activities and co-ordinating involvement. They were also responsible for monitoring changes in capacity of our members living with dementia throughout the trial and supporting their individual needs. Membership did change in the advisory group during the trial due to changes in capacity as some individuals condition deteriorated. A research assistant was also identified to support PPIE activity.

All PPIE activities, records of discussions, the advice given to researchers and how it was subsequently used was recorded on a PPIE activity log (Mathie et al., 2020). This approach allowed us to continually review the impact of advice from our PPIE advisors upon the overall trial and provide transparency and accountability.

## Findings

### Patient and public involvement and engagement in trial oversight and operationalisation

The involvement of people living with dementia was rooted strategically and operationally in Journeying through Dementia trial governance and processes. It was embedded in trial oversight through PPIE membership of our Study Advisory Group and of the independent Trial Steering Committee as well as being included as a standing agenda item for the Trial Management Group. The role of each group/committee was as follows:

• The PPIE Study Advisory Group provided advice and guidance to the academic study team (as described below) using their experiences to inform study design and decision making.
• The Trial Management Group provided operational management of the study. The PPIE lead attended meetings on behalf of the PPIE Study Advisory Group although members could also attend if they wished.
• The Trial Steering Committee, including a PPIE member (who was not a member of the Study Advisory Group, provided independent study supervision, monitored conduct and progress and ensured that the safety and well-being of study participants was upheld (National Institute for Health and Care Excellence, 2019).

**Figure 1.**
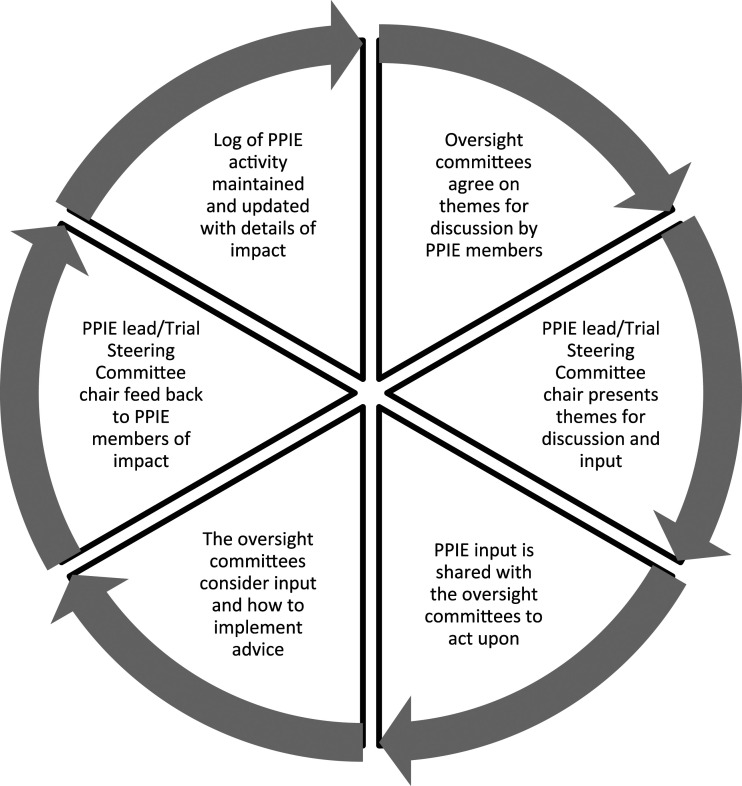
Patient and public involvement and engagement cycle of discussion, implementation and feedback.

As it is important to provide a bridge between PPIE and researchers (Mathie et al., 2020), a cycle of identifying activities, discussion, reporting and taking action was established between PPIE advisors and the management and oversight committees; see Figure 1.

Strategies to support PPIE engagement in the trial tended to be researcher led in the first instance, for example using knowledge of best practice and observation of coping mechanisms of our PPIE members. This was then followed by PPIE member feedback which was used to inform better working practises to support engagement. For example, the research team initially identified dementia friendly venues to hold the PPIE meetings in the community. Difficulties in accessing a venue led to the group discussing other options, as well as improvements to the directions to the venue. Strategies to support engagement were therefore an iterative process throughout the trial as the PPIE group evolved (see Figure 1.).

### Patient and public involvement and engagement Recruitment

Recruitment of PPIE volunteers for membership of the Trial Steering Committee proved challenging. One person living with dementia, who was already active in research and known to the Trial Steering Committee members through existing research networks, was approached to join the Committee ahead of the inaugural meeting and remained a member throughout the four-year trial, attending every meeting. Although the initial intention had been to involve two PPIE members, we found it difficult to recruit a second member to provide cover, support and spread the workload. This may have been due to the four year time commitment, or perceptions of the necessary confidence and skills for this more formal role.

We established a PPIE advisory group to provide advice and guidance to the Trial Management Group throughout, thereby ensuring that our approach remained relevant and accessible to people living with dementia from recruitment to dissemination. Recruitment to the PPIE study advisory group was through the existing University of Bradford ‘Experts by Experience’ cohort of people with a diagnosis of dementia and supporters who volunteer to be involved in research and education (University of Bradford). We recruited individuals who were new to the cohort as well as long-standing members who may have had previous involvement in research. Consequently, some PPIE members had a deeper understanding of research than others. Initially the PPIE lead, and a second member of the research team conducted individual consultations with potential volunteers to explain the trial and what their involvement in the advisory group would entail. They emphasised to each potential volunteer that they could engage in as much or as little as they chose and that involvement in just one event would be valuable. Although interest in joining the group was high, with 10 applicants for the first meeting in July 2017, meeting attendance was initially poor (three people) due to a clash with another meeting held by a local dementia support group. However, the advisory group continued to meet between July 2017 and October 2019 and numbers steadily increased to 12. Several people with dementia attended with a spouse or another family member but others attended alone. Some changes in membership took place over time due to a change in commitments, through illness, and progression of dementia. The group were predominantly white and therefore did not reflect diversity and the experience of dementia by other ethnicities or social groups (Burton et al., 2019). Another limitation was the lack of representativeness of PPIE advisors across the different geographical areas represented in recruitment to the trial. However, the limited geography enabled face-to-face meetings rather than making use of virtual meetings which was not exploited at the time.

### Supporting patient and public involvement and engagement involvement

#### Trial steering committee

The Trial Steering Committee for the Journeying through Dementia trial met twice yearly and involved members from clinical, academic and PPIE backgrounds.

To ensure that our Trial Steering Committee PPIE member was fully informed time was taken by the Chair and/or Trial Manager prior to each meeting to annotate any associated paperwork or preparatory materials. During meetings, the PPIE member sat adjacent to the Chair to facilitate communications. The responsibilities of the Chair were to ensure that the PPIE member understood all discussions throughout, including limiting the use of acronyms and overly technical language by all members and providing time for the PPIE member to consider and respond to an agenda item or question. With these adjustments in place the PPIE member was able to provide unique insights from the perspective of someone living with dementia and made significant contributions in key areas and documented her experiences of being involved in the trial as an adviser in her blog. The following quotes, obtained with verbal consent, from their publicly available blog, ‘Which me am I today’(Mitchell W, 2014) illustrates their contribution and participation in the Trial Steering Committee.

*‘Many of the [meeting] papers were way beyond me but [name] had put a friendly post-it on each one telling me what each paper was about – wonderful idea. Definitely worth a brownie point’.* Trial Steering Committee PPIE member

*‘I raised the question of the reality of relying on our [participants with dementia] answers in follow-ups. And that raised a whole issue of current practices. I said that even if it shows how the current practices need to be revisited, that’s a good outcome. I said revisits 8 months after an event and asking us to recollect is a tad adventurous. We don’t like to feel embarrassed at not remembering so may make things up so we don’t look stupid……or we simply give an answer that comes to mind today’*. Trial Steering Committee PPIE member

#### Advisory group

The operational management of the trial was overseen by the Trial Management Group who agreed that embedding high quality PPIE into protocol development and trial processes throughout was a priority. The PPIE lead for the trial (CM) and coordinator of the PPIE advisory group was a member of the Trial Management Group and PPIE was a standing-item on every meeting agenda.

To ensure that the PPIE advisory group felt supported and integrated into the study team we employed several methods. Firstly, we used our guiding principles to support the establishment and engagement of the group, see Box 1. One to one discussion was offered and taken up by some individuals instead of participation in a group or to aid their decision to join the group. When an advisory group had been assembled, we asked members about their preferences for involvement including how they would like to be communicated with throughout the study, the length and duration of meetings and meeting venues. All members did subsequently take part in the group but some people with dementia needed support from their accompanying supporter to achieve this. In addition, brief verbal and written reminders were provided at every advisory group meeting to reiterate the purpose of the study and what taking part entailed. A welcoming and informal structure was maintained to encourage active participation. At the start and end of each meeting time was scheduled for refreshments, creating opportunities for PPIE advisors to enjoy a group atmosphere, and share more personal experiences and coping strategies whilst at the same time advising the study.

To promote inclusivity, support group dynamics and maintain active interest in the study, all members were invited to be involved in every activity. On most occasions all members attended, reflecting the overall interest the study generated and the possible benefits people were deriving from taking part. For larger groups however, it was challenging to ensure that the voices of all members were heard. PPIE advisors with a diagnosis of dementia could need additional time to process discussions and respond accordingly. This could lead to another person (often their supporter), speaking before them or on their behalf. We therefore used techniques such as turn-taking and signalling using purpose made cards or simply a ‘hands-up’ gesture to try and avoid this.

Dementia friendly, accessible venues in familiar city centre locations were identified by the group in preference to university campus settings which were deemed too busy and confusing. Use of community venues proved positive and some advisory group members reported that they had engaged in an activity in or near such venues following meetings such as shopping or going for lunch. Whether this was simply opportunistic or attending the advisory group resulted in greater confidence to do more activity is unclear.

Activities where the Trial Management Group requested specific input from PPIE advisors and how their contribution impacted the study included:

1. the design of participant newsletters which were originally designed in newspaper column format but were changed to a cross page format which our advisory group reported as being easier for people living with dementia to read.
2. the content of a study proforma completed by facilitators and sent to study participants, summarising the group or individual sessions as part of the intervention. With consultation identified that the content needed to be personalised by adding the participants name and that the overall language used should be simplified using less research terminology.
3. the content, deliverability and impact of burden for our qualitative interview schedule. Advisor feedback proposed that the language and wording should be more concrete for study participants to engage with and that to scaffold recall during the interview researchers should use prompts about what had taken place during intervention sessions. Consequently, the researchers asked shorter more direct questions for clarity and understanding; and referred to activities the participant took part in to prompt recall.

#### Trial delivery and data collection

Our PPIE group advisors’ and Trial Steering Committee PPIE member’s views and ideas on participant retention during the lifetime of the study were invaluable. For the trial we were collecting outcome data for up to 12 months after recruitment from all participants whether they had attended the intervention or been randomised to treatment as usual. Recommendations that were actioned included:

• Emphasising the value of contributions from all participants whether they received the intervention or not in the participant newsletter.
• Booking follow-up researcher visits in advance as part of their first visit with a participant. This would mean that all follow-up visits would be in the diary and a confirmation would just be needed nearer the time of the follow-up.
• Sending a reminder card (rather than letter or sheet) before attending follow-up appointments. PPIE members proposed that a card would be brighter and more visually appealing to participants thereby reducing the anxiety that a formal letter may provoke.
• Including a photo of, and a personal message from, the researcher doing the follow-up.
• Communicating with supporters about the importance of the person with dementia’s participation in the study.

#### Data analysis

Advisory group members used their personal experiences of living with dementia to assist the researchers to understand and interpret our qualitative data.

Advisory group members were approached to participate in two half day validation workshops to discuss and reflect upon researcher interpretations of anonymised data from qualitative interviews conducted with participants and their supporters. Their contributions informed the final analysis of interview data (Sprange et al., 2021). We had to obtain consent from each participant at the outset and identified appropriate researcher resource to do this in a relaxed manner. However, this was could not be achieved with those who arrived late to the workshop where consent was taken at the earliest convenience, typically a break time, so as not to disrupt the flow of the group.

Quotations selected through researcher consensus to be representative of the themes identified in the framework were presented as raw data, i.e. without coding or categorisation (Sprange et al., 2021). These were presented for discussion one at a time alongside some descriptive and contextual detail to aid understanding. For example, who was speaking i.e. participant or supporter and what the person was talking about, such as an element of the intervention or about its facilitation. Consideration was given to how to present each quote to the group in a dementia friendly format to aid understanding (DEEP). This included use of large font size, colour of paper, amount of text per quotation and printing one copy per person. By using ongoing validation observation techniques such as listening and reflecting to gauge understanding and interest in the activity during the workshops the researchers were able to support participation.

The difficulties posed by dementia as experienced by participants were varied which made pitching the task correctly and maintaining the engagement of everyone a challenge for researchers. During our first workshop the level of direction provided by researchers was therefore relatively high. As this was a novel approach to PPIE in dementia research there was some concern expressed by the researchers of not wanting to overwhelm the group. Therefore, different approaches were needed to engage and support those with more severe memory deficits e.g. giving adequate time for the group to read and re-read quotation as well as presenting quotation in both written and verbal formats (facilitator read aloud the quotation). The facilitator also started with an open-ended question such as “what do you think this person is saying/feeling?”, but this may have been followed up with a more structured question to aid contribution for example focussing on an interesting word or phrase in the quotation to initiate discussion. For those with less severe symptoms of dementia, and the supporters who took part, there was enthusiasm and great interest in the research and being part of the interpretation of findings. Those less cognitively able appeared to enjoy the social occasion but it was less clear whether they had been able to engage with all the materials.

Whether to provide participant training for this activity was debated amongst the research team. Some considered that training would be helpful to guide and support engagement in the activity (Roberts et al., 2019), whereas others felt that memory and retention of training prior to the activity may be challenging and therefore could cause frustration or distress. We ultimately decided not to undertake a separate training session prior to the workshops but instead we took time at the start of each session (after consent was taken) to summarise the study and the activity. We included a practice example which we worked through together where the researcher could prompt the group on items we were looking for feedback such as languge used or emotions expressed in the quotation. In addition we also provided visual aids in the form of flip charts bullet stating key facts about the trial to scaffold memory (DEEP).

To support engagement we provided props such as ‘I want to speak please’ cards (DEEP) as a communication aid to indicate when a person wanted to speak. However, we found in our experience that these cards were seldom used. Potential reasons were that firstly, the group were not used to using these props and therefore there was no habit to do so, and secondly this was a well-established group where supporters as well as the more cognitively able members already felt comfortable speaking with each other and enabling each other to participate. However, we also noted that those who were more challenged by symptoms of dementia were more likely to use the cards.

#### Dissemination activity

Advisory group members were consulted on the format and content of both hard copy and online versions of a lay summary of study results. These documents were intended for a wide readership including people living with dementia who had taken part in the trial, members of the public and health and social care professionals. PPIE feedback led to the inclusion of information about organisations that can support people living with dementia and information about how the results might be used to inform healthcare and future research. The group also helped us design and produce a satisfaction questionnaire to obtain feedback regarding the presentation and comprehensibility of the summary findings. This was considered important to review whether our findings were accessible and relevant to people living with dementia and those who care for them as well as for the lay public, clinicians and academics.

Our final trial dissemination event open to all was held in a central public venue with invitees including researchers who had been associated with the study, people living with dementia, members of the public and health and social care professionals. Advisory group members suggested the need for a speaker protocol to encourage presenters to make their session accessible for people living with dementia including means by which those attending might interact with speakers. Consequently, all speakers were provided with a protocol and large cards were made available at the venue that stated, ‘I don’t understand’, ‘I want to ask a question’ and ‘Please speak more slowly’ which delegates did use.

PPIE advisors also recommended that study team members wore brightly coloured sashes that identified them as ‘helpers’ and suggested they should be situated at the main public transport hubs where people attending the event might arrive and at the venue entrance. A member of the advisory group, a former supporter, also volunteered to co-host the event reception desk with a member of the study team. At a previous meeting, advisory group members had been invited to speak about their experiences of being involved in research, but none accepted. Several advisory group members did however volunteer to be involved in making a video to demonstrate the intervention as part of a study dissemination film (https://www.sheffield.ac.uk/scharr/research/centres/ctru/jtd) which was viewed at the event.

## Discussion

We achieved involvement in all stages of this large randomised controlled trial at a time when this level of engagement of people with a dementia diagnosis was not established practice. Importantly the voice of people living with dementia and their supporters was heard first-hand and acted upon, which is acknowledged as being essential (Poland et al., 2019). However, we also found that meaningful involvement could be challenging at times and our aspirations could not always be met, particularly given that PPIE described here was for a trial with necessary study processes such as rigid methods of consenting people into a trial.

During PPIE involvement in data analysis activities we found that the informed consent process could easily disempower people, including those with the capacity to consent. The process was time consuming and burdensome for some people with a diagnosis of dementia who found it confusing to have to agree to numerous statements. It is important that people living with dementia feel empowered to make decisions for themselves when consenting (Thorogood et al., 2018). We therefore suggest that using a simplified consent form, co-produced with people living with dementia, would minimise unnecessary participant burden whilst complying with research governance requirements. At the outset of this trial, researchers aspired to create and test methods of video consent for potential trials participants, but it was quickly realised that this could not be achieved within the resource constraints of the study.

Having reliable methods in place to encourage and capture the impact of involvement activities was greatly facilitated by having a designated PPIE lead, a role now compulsory with some funders such as the UK NIHR. In addition, researchers within the study team who were both knowledgeable and supportive of PPIE was essential. Our experiences underscore the need for researchers to have expertise in working with people living with dementia or that the requisite training and support is provided so that they always take a sensitive and considered approach, enabling involvement in an informed and nuanced manner (Gove et al., 2018). The more we undertake research involving people living with dementia as PPIE advisors or co-researchers the more we learn to pave the way for models of successful participation in research (Roberts et al., 2019).

Patient and public involvement and engagement advisory group members were recruited from existing PPIE cohorts and networks. Whilst this approach perhaps led to a more relaxed exchange of ideas between researchers and PPIE advisors, it created limitations in terms of diversity. Almost all our PPIE advisors were White. Also, some members were living with more advanced stages of dementia which did not reflect the population of study participants. As the membership of the PPIE advisory group evolved, people living in earlier stages of dementia, including some who lived alone joined the group. How to ensure the involvement of the range of people who represent any one group remains a challenge (Witham et al., 2020) and in common with overall recruitment to dementia studies, achieving diversity is difficult (Field et al., 2019). We recommend that to reflect the study population, early liaison, during the design phase of research, with representatives from relevant community organisations might gain support, both for participant recruitment and for recruitment of PPIE representatives. This may in turn increase interest in research from underrepresented groups.

It may require time for people living with dementia to feel participation in research is meaningful (Stevenson & Taylor, 2019). Feeling useful and being able to help others is important to people living with dementia (Perkins R et al., 2013) but it is important to consider what might be other motivations and needs of people acting as PPIE advisors to research. We found that members who had recently received their diagnosis sought personal support and specific advice from the group and from researchers regarding their recent diagnosis and what this meant for their future. Those supporting PPIE therefore need to understand the boundaries between research, clinical advice and personal support and be prepared to respond by signposting individuals to appropriate services. Family members can feel the need to protect people living with dementia, which can lead to a form of gatekeeping, taking decisions on behalf of the person with dementia (Waite et al., 2019). During this study we found that some supporters spoke on behalf of their spouse on occasion. Researchers were aware of the need to listen to the voice of the person with dementia (Gove et al., 2018) and study team members explored ways of achieving this for example ‘turn taking’ and using smaller discussion groups. It has also been observed that offering guidance to supporters on how to enable the people they support to be involved in PPIE activities may be beneficial and make the supporter feel valued (Gove et al., 2018).

Involvement in the Trial Steering Committee for this study did not meet agreed best practice (National Institute for Health and Care Excellence, 2019) in that only one person with a diagnosis of dementia was recruited to the committee. The arrangement was considered to work well due to the skills and previous experience of the PPIE representative but having two members to take account of absence and meet needs for peer support is recommended. Being a Trial Steering Committee PPIE member had different demands compared to the PPIE advisory group member, for example, due to the time lag between meetings and the necessary independence of the Trial Steering Committee which aids objectivity but also creates distance from the study. This can affect ability of all PPIE members to retain knowledge and understanding of the trial, but particularly if the person is living with memory issues. Therefore, approaches to scaffold memory and recall are helpful and should be provided as we identified during this study.

In accord with best practice (Ashcroft et al., 2016; Litherland et al., 2018; Wilson et al., 2015), providing opportunities to share experiences and coping strategies whilst at the same time advising on the study proved important. Additionally, we found that hosting advisory group meetings in a community setting could provide social opportunities that might not have occurred otherwise. Most of our PPIE advisors were, or had recently been, involved in other research studies which perhaps created an understanding of research, and familiarity with other PPIE advisors, that was helpful to enable them to participate.

Our experiences have confirmed that PPIE approaches, and processes need to be established early on to have greatest effect upon the design and implementation of a study. The time taken to establish the advisory group meant that some decisions which would have benefited from PPIE input were initially taken in the absence of consultation, for example, the format and presentation of newsletters sent to study participants which was subsequently changed following recommendations from the advisory group.

Questions remain about how to achieve maximum involvement in research outputs such as presentations and publications. The window within which meaningful engagement can occur is influenced by a complex interaction of factors including the impact of diminishing cognitive abilities, other impairments as a consequence of dementia and the resilience of the individual. However, when working with people with a diagnosis of dementia (as some of our PPIE advisors were) the convention of writing most outputs at the end of a study can limit the participation of PPIE advisors as it relies on recall. Indeed, in the preparation of this paper for publication we left writing up towards the end of the trial and the PPIE advisor approached to contribute felt they could not recall their involvement sufficiently to directly contribute, thus creating disparity between the voice of the researchers and the voice of people living with dementia. We therefore propose assisting PPIE advisors to record their experiences in real time which might result in authentic publication. This would also build up evidence for dissemination or create opportunities for on-project dissemination activities reinforcing the value of the PPIE role and contribution.

Involving people living with dementia in the analysis of data can improve the quality of research, and if done well can be a satisfying experience for PPIE advisors and researchers (Stevenson & Taylor, 2019). The importance of providing PPIE members with appropriate training and support for this and for other aspects of the role is indisputable but questions remain about how this can be achieved to best effect when involving people living with dementia (Roberts et al., 2019). More specifically; for people with a diagnosis of dementia when does PPIE involvement become too much of a challenge (Waite et al., 2019) and who decides; and secondly how can needs for training and support be most effectively met (Wilson et al., 2018).

## Conclusion

We posit that we could improve engagement of people living with dementia in research through increasing diversity and adjusting research processes to be more accessible. This in turn would create parity of voice between people with lived experience and researchers and increase the impact of meaningful PPIE in research whilst improving the experience for PPIE advisors. Many aspects of our approach to involving people living with dementia in supporting study processes were effective in that members of the advisory group reported their involvement as enjoyable, sociable and satisfying. Regular review of the purpose and approach to PPIE on any study is necessary and can improve the experience for PPIE members. There is also a need to be realistic in time people with dementia might be willing to give and any opportunity for shorter commitment with ‘succession planning’ in place.
